# Are Verbal Fluency and Nonliteral Language Comprehension Deficits Related to Depressive Symptoms in Parkinson's Disease?

**DOI:** 10.1155/2012/308501

**Published:** 2012-02-12

**Authors:** Christina Tremblay, Oury Monchi, Carol Hudon, Joël Macoir, Laura Monetta

**Affiliations:** ^1^Département de Réadaptation, Faculté de Médecine, Université Laval, QC, Canada G1V 0A6; ^2^Centre de Recherche Université Laval Robert Giffard, 2601 Rue de la Canardière, Québec, QC, Canada G1J 2G3; ^3^Functional Neuroimaging Unit, Institut Universitaire de Gériatrie de Montréal, Montréal, QC, Canada H3W 1W5; ^4^École de psychologie, Université Laval, QC, Canada G1V 0A6

## Abstract

Depression in Parkinson's disease (PD) is frequently associated with executive deficits, which can influence nonliteral comprehension and lexical access. This study explores whether depressive symptoms in PD modulate verbal fluency and nonliteral language comprehension. Twelve individuals with PD without depressive symptoms, 13 with PD and depressive symptoms (PDDSs), and 13 healthy controls completed a semantic and phonemic verbal fluency task and an indirect speech acts comprehension task. All groups had the same performance in the phonemic fluency task while the PDDS group was impaired in the semantic task. For the indirect speech act comprehension task, no difference was observed between the groups. However, the PDDS group had difficulty answering direct speech act questions. As some language impairments in PD become apparent when depressive symptoms are associated with the disease, it would appear to be important to take the presence of depressive symptoms into account when evaluating language abilities in PD.

## 1. Introduction

The most frequently reported nonmotor symptoms in nondemented individuals with Parkinson's disease (PD) are psychiatric deficits (such as depression, apathy [1], and sleep disturbances [2]) and cognitive impairments (e.g., executive functions [3] and language deficits [4]). Depression, a symptom reported in approximately 27% to 40% of individuals with PD [5], has been shown to increase motor symptoms [1] and cognitive impairments [6], to be associated with more rapid disease progression [7], and to diminish quality of life [8]. As is already known, depressive symptoms are frequently associated with executive deficits [9], which in turn can influence the participant's performance in other cognitive situations such as abilities involving high-level language processing. Indeed, non-literal language comprehension and lexical access (verbal fluency) are largely dependent on executive functions [10]. Non-literal language (such as indirect speech acts, metaphors, or irony) comprehension requires inhibiting and going beyond the literal meaning of a sentence according to the context [11]. As for verbal fluency, this ability involves the retrieval of semantically or phonemically related words from a subcategory (clustering) and then switching to a new subcategory, which requires both mental flexibility and inhibition abilities [12].

Language disabilities are reported in approximately half of individuals with PD. For instance, individuals with PD may have difficulties with semantic [13], syntactic [14], lexical access [15], prosodic [16], and non-literal comprehension abilities [17]. It is well known that PD is heterogeneous in regards to language abilities and other cognitive domains, such as memory or executive deficits. To understand this cognitive heterogeneity, many authors have tried to identify subgroups of individuals with PD principally according to the predominant motor symptoms [18] and the presence or absence of depression [19–21]. Studies have shown that individuals with PD who were depressed had more severe cognitive impairments than others [19]. Many studies have shown an important association between depressive symptoms and cognitive impairments such as executive and memory deficits [9, 22, 23]. Tröster et al. suggested that depression tends to increase the severity of cognitive deficits caused by PD but does not add new ones [24]. The study of Fernandez et al. also supported previous studies results suggesting that depressive symptoms in PD are associated with global cognitive impairments (as demonstrated by the significant correlation observed between BDI and MMSE scores) [25]. However, when they looked at the scores obtained in specific cognitive domains (attention, verbal memory, language, executive functions, and visual-spatial processing) by participants with PD, they found that only verbal memory and language performance (i.e., Hopkins Verbal Learning Test delayed recall and Boston Naming Test scores) showed a significant correlation with depressive symptoms. Thus, one could expect verbal fluency and non-literal language comprehension deficits to be exacerbated by depressive symptoms in PD. If this is the case, treating depressive symptoms in PD may improve specific cognitive and language abilities.

The main purpose of this study was to investigate whether depressive symptoms in nondemented individuals with PD modulate verbal fluency and non-literal language comprehension. Based on previous studies, we anticipated that these two abilities would be problematic for individuals with PD [16, 26], but that performance would be negatively influenced by depressive symptoms.

## 2. Materials and Methods

### 2.1. Participants

Twenty-five individuals diagnosed with idiopathic PD (mean ± SD age:  63 ± 6  years; education:  14 ± 3  years; 9 women and 16 men) participated in this study. The diagnosis of idiopathic PD was made by a movement disorder neurologist on the basis of accepted motor criteria [27]. Motor disability of individuals within the PD group was in the mild-to-moderate severity range (I–III) according to the Hoehn and Yahr (H&Y) staging criteria [28] and was also evaluated with UPDRS-III (Unified Parkinson's Disease Rating Scale part III) (mean ± SD: 29.92 ± 9.05) [29]. In addition to their dopaminergic medication, some participants were taking other medication as follows: venlafaxine 112.5 mg/day (*n* = 1), citalopram 60 mg/day (*n* = 1), irbesartan (*n* = 1), levothyroxine (*n* = 1), rosuvastatin calcium (*n* = 1), amphetamine (*n* = 1), atorvastatin (*n* = 3), warfarine (*n* = 1), folic acid (*n* = 1), sotalol (*n* = 1), hydrochlorothiazide (*n* = 1), and metformin (*n* = 1).

Participants with PD were divided into two subgroups according to their depression level: 12 without depressive symptoms (PD) and 13 with depressive symptoms (PDDS). The Beck Depression Inventory (BDI-II) was used to assess depressive symptoms in all participants [30]. The BDI-II is a 21-question, multiple choice, self-report inventory covering cognitive, behavioral, and somatic aspects of depression. The BDI was selected as the measure of depressive symptoms because of its reliability in identifying depression in older adults [31] and PD individuals [32]. To have the maximum sensibility to screen for depressive symptoms, the cut-off score of 8/9 was used [33].

The two groups of patients with PD were compared to 13 healthy controls (HCs) without depression. Participants with history of alcohol abuse or neurological disease other than depression and PD were systematically excluded. All individuals were screened for dementia prior to the experiment using the Montreal Cognitive Assessment (MoCA) [34]. If their score was <26, they were also excluded of the study, but none of the participants met the criteria for dementia. All participants had normal or corrected-to-normal vision as determined by self-report.

### 2.2. Materials and Procedure

An informed consent was obtained for experimentation with human subject and participants were compensated for their participation. Tests were approved by the ethics committee of the University of Montreal and administered according to these committee guidelines. The privacy rights of each participant were always observed. Participants were evaluated individually in a quiet room during a two-hour session. As performance on cognitive and language tasks is typically influenced by Levodopa therapy [35], all participants with PD were evaluated while they were off their dopaminergic medications (at least 12 hours following withdrawal). All tests, including H&Y, UPDRS-III, BDI-II, and MoCA, were performed without dopaminergic medications.

### 2.3. Neuropsychological Tests

Participants completed a battery of standardized neuropsychological tests, which included measures estimating executive resource functions. Three different measures linked to executive dysfunctions generally found in PD were used: processing speed, planning, and inhibition. To measure processing speed, the first subtask of the Stroop Color Word Test [36] and the Trail-Making Test A [37] were used. In the Stroop Color Word Test (subtask 1), the time required to read 100 color names appearing in black on a sheet was recorded and the scores were then converted in the number of words said in 45 sec. The time make to complete the part A of the task was also recorded in the Trail Making Test to measure the processing speed. The number of errors in the Tower of London test [38] was used to measure planning. The inhibition score was evaluated with the Hayling test part B [39] and the third subtask of the Stroop Color Word Test (number of errors in both cases). The error score in the Stroop Color Word Test was calculated as the number of errors in the inhibition condition (subtask 3) minus the number of errors in the control condition. Moreover, the Brixton Spatial Anticipation Test was also used to measure anticipation, mental flexibility, and set-shifting [40].

### 2.4. Language Tasks

Participants completed two tasks evaluating language abilities.

#### 2.4.1. Verbal Fluency Tasks

Participants were evaluated on a semantic and a phonemic verbal fluency task from the Montreal Evaluation of Communication (MEC) protocol [41]. For the phonemic verbal fluency task, participants had to say as many words as possible starting with the letter “F” in two minutes. Proper names, repeated words, and morphologically derived words were rejected. For the semantic verbal fluency task, participants had to name as many items of clothing as possible in two minutes. The number of words said in this lap of time was recorded and analyzed in both tasks.

#### 2.4.2. Nonliteral Language Comprehension Task

An indirect speech act task from the MEC Protocol [41] was used to evaluate non-literal language comprehension. This task includes short stories that refer to everyday-life situations where the intention of the person is not explicitly mentioned with reference to a context (e.g., “John is in his bedroom listening to music. His dad tells him: John, your door is open. What do you think his father means?”), or where the person says explicitly what they wanted to say (e.g., “Mr. Larsen works in an office and is printing a document. He says to his secretary: This printer is very efficient. What do you think Mr. Larsen means?”). An explanation and an example of questions with indirect and direct speech acts were given verbally by the experimenter to familiarize participants with the procedure before beginning the test. Participants were instructed to listen to the stories and respond verbally to the questions. Twenty stories (half indirect and half direct) were presented to each individual in a random order.

### 2.5. Statistical Analyses

Taking into account the normality of data distribution, the analyses were conducted using ANOVA and the Tukey or the Games-Howell post hoc tests to compare the data between the three groups. Mann-Whitney test was performed for dichotomous variables. Relationships between variables were investigated with the Spearman rank correlation test because the indirect speech act scores were not normally distributed. All raw scores were converted to *z*-scores, taking into account the mean and the standard deviation of the control group scores, to calculate correlations. The results were considered significant if *P* < 0.05. As it is known that antidepressant drugs could influence individual's cognitive performance, all analyses were also performed excluding the two participants that were taking medication for depression (venlafaxine and citalopram). As no difference was found between the significativity of the results with or without these participants, both participants were included in the present statistical analysis.

## 3. Results

### 3.1. Demographic and Clinical Data

Table 1 reports the clinical and sociodemographic characteristics of the participants. In the present study, the HC and PD groups did not have significant depressive symptoms (BDI scores between 0 and 7) while the PDDS group had mild-moderate depressive symptoms (BDI: 10 to 28), a highly significant difference (*F*(2,35) = 40.77, *P* < 0.001). The PD and PDDS groups were comparable on age (*F*(2,35) = 1.48, *P* = 0.24) and education (*F*(2,34) = 1.12, *P* = 0.34) with the HC group. The two patients' groups were also comparable on disease duration (*P* = 0.22), age at disease onset (*P* = 0.69), and UPDRS-III (*P* = 0.17), factors known to influence cognitive abilities in PD [42–44].

The results for verbal fluency, non-literal comprehension, and on the neuropsychological tests are reported separately in the next three subsections.

### 3.2. Verbal Fluency

**Figure 1:**
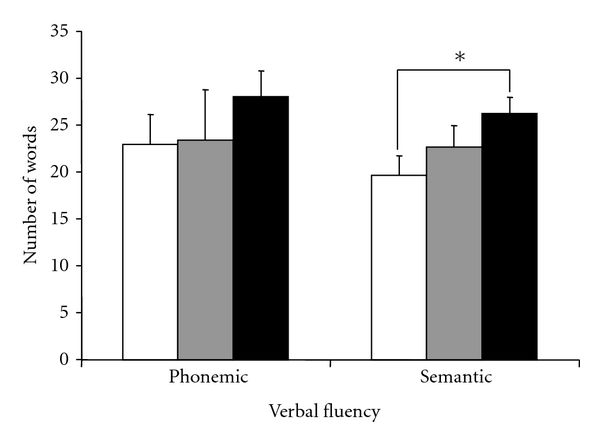
Phonemic and semantic verbal fluency mean results (±SEM) for participants with Parkinson's disease with (white column) and without (grey column) depressive symptoms and healthy controls (black column).

Figure 1 presents the mean numbers of words produced in the phonemic and semantic verbal fluency tasks for the HC group and both PD groups. The ANOVA revealed a significant difference between the three groups (PD, PDDS, HC) for semantic verbal fluency (*F*(2,34) = 3.64, *P* = 0.04). Interestingly, the scores of PDDS in this task were significantly different than those of the HC group (*P* = 0.03) whilst there was no significant difference in the number of words produced by HC and PD participants (*P* = 0.28) as indicated by post hoc comparisons using Tukey test. In the phonemic verbal fluency task, there was no significant difference between the HC and the two PD groups (*F*(2,34) = 98.23, *P* = 0.15).

### 3.3. Nonliteral Language Comprehension

**Figure 2:**
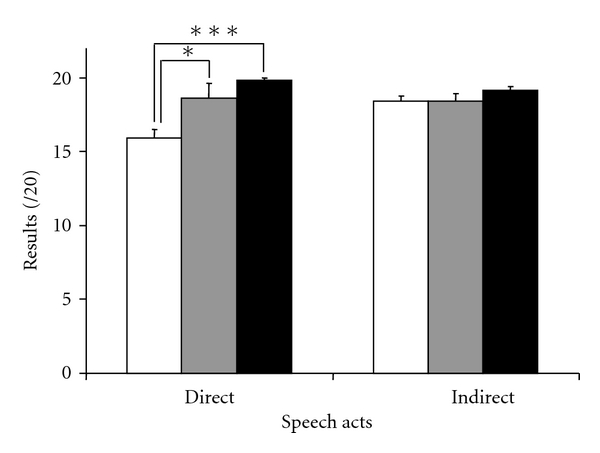
Direct and indirect speech act mean results (±SEM) for participants with Parkinson's disease with (white column) and without (grey column) depressive symptoms and healthy controls (black column).

Figure 2 reports the accuracy in answering direct and indirect questions for the three groups of participants. There were no significant differences for indirect speech acts between the three groups (*F*(2,34) = 1.21, *P* = 0.31). However, the ANOVA conducted to compare the mean scores for direct speech acts revealed a significant difference between the three groups (*F*(2,35) = 9.61, *P* < 0.001). Furthermore, post hoc comparisons using the Tukey test indicated that the PDDS group's direct speech act results were significantly lower than the scores of both PD (*P* = 0.02) and HC (*P* < 0.001) groups. The mean score of PD and HC was not significantly different (*P* = 0.38).

### 3.4. Neuropsychological Tests and Their Correlation with Language

Table 2 presents the neuropsychological performance of healthy controls, PDDS, and PD participants. Statistical comparisons performed on the neuropsychological tests results of the three groups revealed only one significant difference between PDDS and PD, and PDDS and HC on the Tower of London test (number of errors) (*F*(2,35) = 29.71, *P* = 0.003). The Games-Howell post hoc test indicated that the PDDS group made significantly more errors than the PD group (*P* = 0.047). Moreover, this post hoc test revealed a significant difference on the Tower of London scores (number of errors) between PDDS and HC groups (*P* = 0.04), while there was no difference between PD and HC (*P* = 0.87). Taken together, these results indicate that Parkinson's disease with depressive symptoms has a negative effect on planning ability (as evaluated by the Tower of London test), but that Parkinson's disease itself has no effect on this capacity. Scores obtained by the PD and PDDS groups were similar to HC scores on the Stroop (number of words and number of errors) test, the Color Trail-Making Test A, and the Hayling test, evaluating inhibition and processing speed. However, statistical analysis revealed a significant difference on the Brixton Spatial Anticipation Test between groups (*F*(2,35) = 4.90, *P* = 0.01). Post hoc comparisons using the Tukey test indicated that the mean score for the Brixton Spatial Anticipation Test was significantly different between PDDS and HC groups (*P* = 0.02) as well as between PD and HC participants (*P* = 0.03).

To verify if these executive impairments affected performance in language tasks, correlations between direct speech acts, semantic verbal fluency results, and selected composite scores were calculated. Prior to the analyses, raw data from the neuropsychological tests were transformed into *z* scores, taking into account the control group performance. Then composite scores were calculated to estimate the patients' capacities in processing speed (performance in the first subtask of the Stroop [number of words] and Color Trail-Making Test A [time]) and inhibition (performance in the third subtask of the Stroop [number of errors] and in the part B of the Hayling [number of errors]). No composite score was calculated for estimate the participants' abilities in planning because only one test was taken into account (performance in the Tower of London [number of errors]). Spearman's test showed that the direct speech act results were significantly correlated with inhibition for PDDS participants only (*r*(7) = 0.64, *P* = 0.03). Moreover, a significant correlation was found between the number of words produced in the semantic verbal fluency task and processing speed only for the PDDS group (*r*(7) = 0.85, *P* = 0.002).

### 3.5. Correlations between Clinical Features

Correlations between language test results (direct and indirect speech act scores, and phonemic and semantic verbal fluency scores) and demographic characteristics (age, education, gender, disease duration, age at onset, MoCA, UPDRS-III, and BDI) were investigated for HC, PD, and PDDS groups. Only one significant correlation was found for the PDDS group, in this case between the direct speech act results and BDI scores (*r*(11) = −0.59, *P* = 0.03), showing the influence of depressive symptoms on the interpretation of direct speech acts. Even if the percentage of men differed significantly between HC and the PDDS groups (see Table 1), no significant correlation was found between gender and language test results for both groups, suggesting that gender did not influence the language abilities evaluated. In fact, no significant correlation was found between language test results and all demographic characteristics for both HC and PD groups.

## 4. Discussion

The goal of this study was to investigate whether depressive symptoms are associated with different patterns of performance on verbal fluency and non-literal language comprehension tasks in nondemented individuals with PD. No difference was observed between HC, PD, and PDDS groups in the phonemic fluency task while only the PDDS group was impaired in the semantic fluency task. An association was observed between the number of words produced in this task and the processing speed scores. Moreover, all groups had the same performance in the indirect speech act comprehension task, but only the PDDS group had difficulty answering direct speech act questions. Interestingly, PDDS group scores on direct speech act questions were only associated with inhibition abilities. These findings support Fernandez et al. study showing that while depressive symptoms correlate with global cognitive performance, language abilities seem to be the most susceptible cognitive domains affected by depressive symptoms [25].

### 4.1. Verbal Fluency

With respect to verbal fluency tasks, results showed that both PD subgroups had a good performance for phonemic fluency. Although there are controversial results in the literature about phonemic fluency in individuals with PD [45, 46], our results are in agreement with most of the previous studies [26, 47], suggesting that PD with or without depressive symptoms do not impair the phonemic verbal fluency capacities. However, our results showed that only the PDDS group was impaired on the semantic fluency task while the PD group's performance was comparable to the HC group. Most of the literature showed impaired semantic fluency for nondemented individuals with PD [15, 26, 47], but mild-to-moderate depressive symptoms were not taken into account in these researches. Our results thus suggested that semantic fluency is modulated by depressive symptoms while phonemic fluency is not, which is somewhat odd because depressive symptoms are generally associated with executive deficits [25] and phonemic fluency is known to require more executive capacities than semantic fluency [14]. One possible explanation may be that these PD patients have particular semantic deficits in addition to specific cognitive deficits (such as processing speed decrement, as supported by the significant correlation found between the number of words produced in the semantic fluency task and the processing speed scores) leading to more difficulties to quickly find words semantically related. Indeed, Portin et al. found that only PD patients with mild cognitive deficits have a storage impairment of semantic knowledge [48]. They observed that PD participants with mild cognitive impairments performed worse than PD patients without cognitive deficits in defining concrete and abstract concepts and in tasks demanding evaluation of hierarchical semantic relations. However, other investigations exploring the impact of different cognitive deficits combined to specific semantic impairments on verbal fluency in PD would be necessary to confirm this hypothesis.

### 4.2. Nonliteral Language Comprehension

Comprehension of indirect speech acts in individuals with PD was also evaluated since previous studies showed that more than half of them presented non-literal language understanding deficits [16, 49]. No significant difference was observed for indirect speech act comprehension between the scores of PD, PDDS, and HC. So neither PD nor mild-to-moderate depressive symptoms in PD seemed to have any influence on indirect speech act comprehension in this offline task. In an online experiment, McNamara et al. observed an inefficient activation of indirect meaning of indirect speech acts and a correlation with participants' executive dysfunctions [50]. However, in a second experiment conducted offline by the same researchers, no significant difference was showed for indirect speech acts comprehension between PD and control groups. McNamara et al. concluded that PD and control did not differ in their interpretation of indirect speech acts, but that activation speed of indirect meanings was slower for PD participants than for control. Our results are consistent with the offline results of McNamara's study, indicating no impairment in indirect speech act comprehension for the PD participants, whether or not they show depressive symptoms.

 Surprisingly, our results showed a significant difference in direct speech act scores between HC and PDDS participants. We found that only the PDDS group had difficulty answering direct speech act questions while the PD group performed like the HC individuals. This unexpected result was also found in previous study with a right-hemisphere-damaged population [51]. Researchers have suggested that participants' impairment in the direct speech acts was caused by their inhibition deficit preventing them from inhibiting irrelevant information and leading the participants to overattribute intention to the protagonist and to understand direct speech acts as indirect ones. As we found in the present study that inhibition scores correlated significantly with PDDS direct speech act results, this explanation is also applicable for the PDDS participants. However, further investigations with more participants would be necessary to confirm this hypothesis.

 As previously presented, this study showed that semantic fluency and planning ability in PD become apparent or more severe when depressive symptoms are associated with the disease (as shown by the fluency and the Tower of London results, resp.). These findings suggested that depressive symptoms in PD would be related to specific neuropathological changes. Indeed, many studies argue that depression in PD seems to result from a degeneration of neurological pathways (including serotoninergic and dopaminergic neurons) that regulate behavior, cognition, and language [21]. One can also argue that cognitive deficits (including language impairments) found in PD with depressive symptoms are just a manifestation of depression. In fact, Kuzis et al. (1997) evaluated PDDS individuals and individuals with depression (MD) but without PD with a comprehensive neuropsychological and psychiatric assessment [23]. They reported that PDDS participants had a worse performance than MD individuals on specific cognitive tasks such as concept formation and switching abilities. Their results showed that cognitive deficits in PDDS group were not only associated with depression but may be related to specific neurological impairments associated with depression in PD.

 Some limitations of the present study deserve mention: (1) the results of this study are only applicable to PD patients off medication and should not be generalized directly to PD patients on medication: other studies are necessary to verify if medication is an important factor when investigating the association between language and depressive symptoms in PD; (2) we have a limited number of participants in each group; thus further investigations with more participants would be necessary to generalized the results of this pilot study to population with PD; (3) to better understand the role of depression on different cognitive abilities (including language capacities), it would have been interesting to have a control group with depressive symptoms without PD: further studies on this topic may help to specify which cognitive deficits are particularly associated with depression and which ones are associated with depression and PD.

## 5. Conclusions

In summary, the results of this present investigation are in accordance with many studies indicating that depressive symptoms are negatively associated with cognition in PD. However, still few studies have explored the relationship between depressive symptoms and specific language impairments in PD (e.g., verbal fluency and non-literal language deficits). Among these studies, the present one is the first to explore the associations between depressive symptoms, cognitive capacities (such as processing speed and inhibition), and the performance on specific language tasks in PD. Indeed, in PD individuals, depression is generally associated with memory and executive deficits, which can influence language abilities. It would therefore be very important to take the presence of depressive symptoms into account when evaluating cognitive and language abilities in individuals with PD. Separately evaluating PD and PDDS patients would then be an adequate way of taking into account their differences. This would help, for example, to understand what cognitive or language impairments are caused by PD and which are associated to depression in PD. Furthermore, other studies evaluating semantic, lexical, and syntactic abilities would be very useful to specify what specific language abilities are associated with depressive symptoms.

## Figures and Tables

**Table 1:** Demographic and clinical characteristics of healthy controls (HCs) and participants with Parkinson's disease with (PDDS) and without (PD) depressive symptoms.

Features	Groups			Significant difference with HC
	HC Mean (SD)	PD Mean (SD)	PDDS Mean (SD)	
Age (years)	61,94 (5,62)	61,81 (4,81)	64,77 (6,00)	NS^a^
Education (years)	14,85 (3,37)	13,00 (2,72)	14,23 (2,77)	NS
Gender (% men)	30,77	50,00	76,92	PDDS*
Disease duration (years)	—	4,42 (3,00)	6,31 (4,25)	NS
Age at onset (years)	—	57,39 (6,86)	58,46 (6,32)	NS
Montreal Cognitive Assessment (MoCA)	28,38 (1,27)	28,08 (1,16)	27,15 (1,77)	NS
UPDRS-III	—	26,58 (7,88)	33,00 (9,24)	NS
Beck Depression Inventory (BDI)	2,92 (2,25)	4,17 (2,12)	14,54 (5,32)	PDDS***

All tests were performed without dopaminergic medication.

^a^NS (non-significant); *P < 0.05; ***P < 0.001.

No results are significantly different between PDDS and PD except for BDI (*P* < 0.001).

UPDRS: Unified Parkinson's Disease Rating Scale.

**Table 2:** Neuropsychological performance of healthy controls (HCs) and participants with Parkinson's disease with (PDDS) and without (PD) depressive symptoms.

Tests of executive resources	Groups			Significant difference with HC
	HC Mean (SD)	PD Mean (SD)	PDDS Mean (SD)	
Stroop color word test (number of words)	48,77 (10,57)	48,64 (6,64)	44,18 (7,40)	^ a^NS
Stroop color word test (number of errors)	0,38 (0,77)	1,36 (1,57)	1,17 (1,53)	NS
Color trail-making test A	34,08 (8,98)	36,10 (13,76)	44,85 (17,44)	NS
Hayling	4,58 (1,88)	3,73 (1,74)	3,83 (1,64)	NS
London tower (number of errors)	0,46 (0,52)	0,58 (0,67)	3,15 (3,39)	PDDS*
Brixton spatial anticipation test	7,00 (2,27)	4,75 (2,01)	4,69 (2,10)	PDDS*, PD*

All tests were performed without dopaminergic medication.

^a^NS (non-significant); **P < 0.01; *P < 0.05.

No results are significantly different between PDDS and PD except for the Tower of London (number of errors; *P* < 0.05).
